# Comprehensive genome‑wide analysis of the chicken heat shock protein family: identification, genomic organization, and expression profiles in indigenous chicken with highly pathogenic avian influenza infection

**DOI:** 10.1186/s12864-023-09908-y

**Published:** 2023-12-20

**Authors:** Anh Duc Truong, Ha Thi Thanh Tran, Nhu Thi Chu, Huyen Thi Nguyen, Lanh Phan, Hoai Thi Phan, Thi Hao Vu, Ki-Duk Song, Hyun S. Lillehoj, Yeong Ho Hong, Hoang Vu Dang

**Affiliations:** 1 Department of Biochemistry and Immunology, National Institute of Veterinary Research, 86 Truong Chinh, Dong Da, Ha Noi, 100000 Vietnam; 2 The Animal Molecular Genetics and Breeding Center, Department of Animal Biotechnology, JeonBuk National University, Jeonju, 54896 Republic of Korea; 3 Department of Animal Science and Technology, Chung-Ang University, Anseong, 17546 Republic of Korea; 4 Animal Biosciences and Biotechnology Laboratory, Agricultural Research Services, United States Department of Agriculture, Beltsville, MD 20705 USA

**Keywords:** Chicken, Heat shock proteins, HSP genes, Genome-wide analysis, Expression profile

## Abstract

**Background:**

Heat shock proteins (HSPs) function as molecular chaperones with critical roles in chicken embryogenesis, immune response to infectious diseases, and response to various environmental stresses. However, little is known on HSP genes in chicken. In this study, to understand the roles of chicken HSPs, we performed genome-wide identification, expression, and functional analyses of the HSP family genes in chicken.

**Results:**

A total of 76 HSP genes were identified in the chicken genome, which were further classified into eight distinct groups (I-VIII) based on phylogenetic tree analysis. The gene-structure analysis revealed that the members of each clade had the same or similar exon-intron structures. Chromosome mapping suggested that HSP genes were widely dispersed across the chicken genome, except in chromosomes 16, 18, 22, 25, 26, and 28–32, which lacked chicken HSP genes. On the other hand, the interactions among chicken HSPs were limited, indicating that the remaining functions of HSPs could be investigated in chicken. Moreover, KEGG pathway analysis showed that the HSP gene family was involved in the regulation of heat stress, apoptotic, intracellular signaling, and immune response pathways. Finally, RNA sequencing data revealed that, of the 76 chicken HSP genes, 46 were differentially expressed at 21 different growth stages in chicken embryos, and 72 were differentially expressed on post-infection day 3 in two indigenous Ri chicken lines infected with highly pathogenic avian influenza.

**Conclusions:**

This study provides significant insights into the potential functions of HSPs in chicken, including the regulation of apoptosis, heat stress, chaperone activity, intracellular signaling, and immune response to infectious diseases.

## Background

Heat shock proteins (HSPs) or stress proteins are a class of molecular chaperones that play important roles in protein folding and assembly, immune response, intracellular transport/sorting of proteins, control of cell-cycle and signaling, protection against stress, and apoptosis [1]. HSPs are constitutively produced by cells or induced in response to high temperature, hypoxia, infectious diseases, toxins, and several other forms of stress or stimuli [2]. Several HSP genes are utilized as housekeeping proteins with high expression levels in non-stressed cells [2]. HSPs play crucial roles in restoring unfolded proteins to their functional three-dimensional structure under stress conditions [1]. Based on their molecular weight, HSPs are classified into major families including HSP105/110, HSP90, HSP70, HSP60, HSP40 (DNAJ), HSP10, and small HSPs [1, 3–6].

HSP70 proteins, first discovered and characterized in the early 1960s [4], function as the central components of the cellular network of molecular chaperones and folding catalysts [7]. The diverse biological function of the HSP70 gene family, which are well characterized in chickens such as the assembly of multimeric protein complexes, facilitating the intracellular folding of proteins, protein transportation across membranes, and the regulatory responses to the heat shock [8–10]. The expression of HSP70 proteins can be rapidly upregulated in response to environmental stress [11, 12]; the levels of HSP70 family proteins are dynamically regulated upon exposure to extrinsic (pathogenic and environmental) or intrinsic stimuli (physiological, replicative, or oncogenic) [13–15].

J-proteins, also called HSP40, J-domain, or DNAJ-proteins, were initially characterized in *E. coli* as 41-kDa HSPs [5]. The DNAJ-protein, the defining feature of J-proteins, is a compact tetrahelical domain of approximately 70 amino acids with a highly conserved and functionally critical histidine, proline, and aspartic acid tripeptide (HPD) motif [16]. In the HSP70-HSP40 co-chaperone system, the association between HSP70 proteins and their substrates requires the binding of an ATP to the ATPase domain, which is hydrolyzed to change the conformation of the binding domain. Thus, several HSP70 substrates can specifically bind to its least conserved C-terminal with a high affinity. However, because the ATPase activity of HSP70s is extremely weak, the J-domain of HSP40s is needed for activating the ATPase domain of HSP70s [15–18].

HSP90 proteins (inducible HSP-α and constitutive HSP-β) [7, 19] are found in the cytosol, endoplasmic reticulum, chloroplast, and mitochondria to assist the folding, intracellular transport, maintenance, and degradation of proteins as well as participating in cellular signaling and immune responses to pathogenic stimuli by regulating signaling pathways such as PI3K/AKT, VEGF, and NF-kB pathways [3, 20, 21]. Small heat shock proteins (sHSPs; 15–30 kDa) are one of the five types of molecular chaperones that confer tolerance to heat stress. They are found in all domains of life [3, 22] and function to prevent irreversible protein aggregation by binding, rather than refolding, misfolded proteins [23].

However, some recently reported chicken HSPs are still not well-defined; their gene structures, physicochemical properties, and phylogenetic relationships are unknown. In addition, the expression patterns of chicken HSP genes during highly pathogenic avian influenza virus (HPAI) infection remain elusive. In this study, we investigated and characterized all potential HSP genes in chicken, revealing the chromosomal locations, gene structures, and physicochemical properties of chicken HSPs. We also examined the expression levels of chicken HSP genes at different stages of embryonic growth and under conditions of HPAIV infection in two indigenous Ri chicken lines in Vietnam.

## Results

### Identification of potential chicken HSPs genes

A total of 76 HSP genes were identified in chicken genome: 13 genes encoding small HSP family proteins; 44 HSP40 family; one HSP60 family; eight HSP70 family; one HSP47 family; three HSP90 family; three HSP105/110 family; and three HSF family (Table 1). Among these 76 HSP genes, 35 were novel genes that have not been included in databases such as chDNAJA2-4, chDNAJB1-B5, chDNAJC19-23, chHSP2-8, chHSPA4, and chHSPA14 (Table 1). The full-length coding sequences of these genes were found in both transcriptome and genome databases. The predicted molecular weights varied from 10.95 kDa (chHSPE1) to 520.759 kDa (chDNAJC29). The chHSP10 gene had the shortest conserved domain with 102 amino acids, and the longest domain with 4580 amino acids was found in the genes chHSPE1 and chDNAJC29.

**Table 1:** Summary of the chicken HSP gene families

Align	Gene name	Accession Number		Gene		Protein			Genome	
		mRNA	Protein	mRNA (bp)	CDS (bp)	Length (aa)	PI	Mol (kDa)	Located	Gene length
	**DnaJ heat shock protein family (Hsp40)**									
1	chDNAJA1	NM_001012945	NP_001012963	2,602	1,194	397	6.47	44.58	ChrZ (70,798,137–70,805,868)	7,732
2	chDNAJA2	NM_001005841	NP_001005841	1,558	1,236	411	5.8	45.51	Chr11 (7,714,288–7,724,790)	10,503
3	chDNAJA3	XM_025155347	XP_025011115	2,069	1,521	506	9.2	54.79	Chr14 (13,132,313–13,144,401)	12,089
4	chDNAJA4	XM_413746	XP_413746	1,768	1,197	398	5.58	44.67	Chr10 (4,188,977–4,194,476)	5,500
5	chDNAJB1	XM_003643562	XP_003643610	1,159	720	239	7.65	26.73	ChrUn (874,523–880,008)	5,486
6	chDNAJB2	XM_015290094	XP_015145580	1,479	849	282	4.84	30.96	Chr07 (22,194,045–22,198,512)	4,468
7	chDNAJB4	XM_004936669	XP_004936726	4,578	1,020	339	8.16	38.15	Chr08 (19,217,370–19,242,757)	25,388
8	chDNAJB5	XM_004937116	XP_004937173	3,245	1,149	382	8.59	43.14	ChrZ (8,522,820–8,537,948)	15,129
9	chDNAJB6	NM_001012556	NP_001012574	2,641	981	326	8.16	36.54	Chr02 (8,738,313–8,788,235)	49,923
10	chDNAJB8	XM_001233012	XP_001233013	1,234	627	208	6.45	23.72	Chr12 (9,657,256–9,658,489)	1,234
11	chDNAJB9	NM_001030735	NP_001025906	3,266	651	216	7.9	22.79	Chr01 (28,562,682–28,571,177)	8,496
12	chDNAJB11	XM_422682	XP_422682	1,555	1,077	358	5.7	40.25	Chr09 (4,531,049–4,542,952)	11,904
13	chDNAJB12	NM_001031224	NP_001026395	2,966	1,125	374	8.3	42.22	Chr06 (12,152,738–12,167,831)	15,094
14	chDNAJB13	XM_417251	XP_417251	1,167	951	316	6.45	35.54	Chr01 (196,119,550–196,123,957)	4,408
15	chDNAJB14	NM_001031375	NP_001026546	3,810	1,143	380	8.19	42.57	Chr04 (60,128,048–60,153,671)	25,624
16	chDNAJC1	XM_015282022	XP_015137508	2,575	1,623	540	7.83	62.60	Chr02 (17,823,830–17,917,737)	93,908
17	chDNAJC2	NM_001199325	NP_001186254	1,992	1,860	619	7.73	71.29	Chr01 (13,115,336–13,135,310)	19,975
18	chDNAJC3	NM_001008437	NP_001008437	5,053	1,512	503	5.54	54.18	Chr01 (147,369,992–147,401,864)	31,873
19	chDNAJC5	NM_001278008	NP_001264937	1,495	597	198	4.83	21.97	Chr20 (9,520,439–9,536,619)	16,181
20	chDNAJC6	NM_001177415	NP_001170886	4,913	2,748	915	6.41	99.40	Chr08 (28,627,625–28,668,296)	40,672
21	chDNAJC7	NM_001031502	NP_001026673	5,508	1,491	496	5.97	56.34	Chr27 (7,558,532–7,577,845)	19,314
22	chDNAJC8	XM_015297581	XP_015153067	1,463	753	250	8.25	29.48	Chr23 (1,641,840–1,655,689)	13,850
23	chDNAJC9	NM_001199525	NP_001186454	1,702	783	260	5.86	29.71	Chr06 (5,926,431–5,929,549)	3,119
24	chDNAJC10	XM_421968	XP_421968	3,691	2,394	797	6.19	91.37	Chr07 (13,876,663–13,897,103)	20,441
25	chDNAJC11	XM_015296996	XP_015152482	3,119	1,566	521	8.19	58.92	Chr21 (539,938–556,353)	16,416
26	chDNAJC12	NM_001199530	NP_001186459	1,762	591	196	4.92	22.83	Chr06 (7,337,888–7,349,978)	12,091
27	chDNAJC13	XM_004939445	XP_004939502	7,702	6,738	2,245	6.05	254.96	Chr02 (42,192,068–42,245,867)	53,800
28	chDNAJC14	XM_001232018	XP_001232019	684	624	207	6.62	25.12	Chr02 (72,072,957–72,078,593)	5,637
29	chDNAJC15	NM_001277776	NP_001264705	895	447	148	9.52	15.84	Chr01 (168,054,028–168,078,478)	24,451
30	chDNAJC16	NM_001039330	NP_001034419	4,102	2,334	777	6.19	87.12	Chr21 (4,915,864–4,924,565)	8,702
31	chDNAJC17	NM_001267575	NP_001254504	1,070	996	331	8.04	35.20	Chr05 (1,233,060–1,249,381)	16,322
32	chDNAJC18	NM_001025609	NP_001020780	1,045	891	296	6.01	34.59	Chr13 (3,168,035–3,179,689)	11,655
33	chDNAJC19	XM_003641772	XP_003641820	888	336	111	9.5	12.11	Chr09 (17,241,668–17,245,149)	3,482
34	chDNAJC20	XM_003643562	XP_003642255	758	693	230	7.38	26.23	Chr15 (7,979,323–7,982,938)	3,616
35	chDNAJC21	XM_004937181	XP_004937238	3,005	1,611	536	5.51	62.48	ChrZ (10,568,012–10,583,520)	15,509
36	chDNAJC22	XM_025145645	XP_025001413	1,290	906	301	10.9	32.27	Chr33 (5,440,960–5,448,035)	7,076
37	chDNAJC23	XM_004940340	XP_004940397	2,667	2,307	768	5.25	88.85	Chr03 (67,569,395–67,624,889)	55,495
38	chDNAJC24	NM_001190896	NP_001177825	744	441	146	4.66	16.79	Chr05 (5,240,479–5,272,375)	31,897
39	chDNAJC25	XM_003643082	XP_003643130	1,936	1,041	346	8.46	41.46	ChrZ (66,600,760–66,608,122)	7,363
40	chDNAJC26	XM_424873	XP_424873.4	9,296	3,957	1,318	5.27	145.59	Chr02 (53,452,577–53,522,927)	70,351
41	chDNAJC27	NM_213558	NP_998723	822	822	273	7.93	30.65	Chr03 (105,433,713–105,439,944)	6,232
42	chDNAJC28	XM_004934505	XP_004934562	1,426	1,122	373	8.54	43.20	Chr01 (106,666,788–106,669,691)	2,904
43	chDNAJC29	XM_004938796	XP_004938853	16,476	13,743	4,580	5.97	520.76	Chr01 (178,925,593–178,983,820)	58,228
44	chDNAJC30	XM_003642377	XP_003642425	2,568	621	206	11.01	22.26	Chr19 (278,976–281,651)	2,676
	**HSP 70 protein**									
45	chHSPA2	NM_001006685	NP_001006686	2,323	1,905	634	5.4	69.62	Chr05 (53,058,059–53,060,378)	2,320
46	chHSPA4	XM_414655	XP_414655	4,067	2,523	840	5.16	94.11	Chr13 (17,844,032–17,867,251)	23,220
47	chHSPA4L	NM_001012576	NP_001012594	3,130	2,532	843	5.16	94.68	Chr04 (34,300,857–34,323,340)	29,228
48	chHSPA5	NM_205491	NP_990822	2,389	1,959	652	5.03	70.32	Chr17 (9,890,854–9,894,906)	5,269
49	chHSPA8	NM_205003	NP_990334	2,119	1,941	646	5.33	70.70	Chr24 (3,102,900–3,107,842)	6,425
50	chHSPA9	NM_001006147	NP_001006147	3,084	2,028	675	5.89	68.15	Chr13 (3,514,654–3,536,259)	28,088
51	chHSPA13	NM_001030793	NP_001025964	4,193	1,419	472	6.04	50.00	Chr01 (99,458,895–99,466,501,)	9,889
52	chHSPA14	XM_416996	XP_416996	2,125	1,518	505	5.07	53.75	Chr01 (7,970,741–7,981,599)	14,117
	**Heat shock protein family D (Hsp60)**									
53	chHSPD1	NM_001012916	NP_001012934	2,076	1,722	573	5.6	57.99	Chr07 (10,221,920–10,231,186)	12,047
	**Heat shock protein 90 families (Hsp90)**									
54	chHSP90AA1	NM_001109785	NP_001103255	2,187	2,187	728	4.92	83.93	Chr05 (49,679,064–49,684,241)	6,732
55	chHSP90AB1	NM_206959	NP_996842	2,633	2,178	725	4.86	83.30	Chr03 (30,393,089–30,399,047)	7,747
56	chHSP90B1	NM_204289	NP_989620	2,733	2,388	795	4.75	89.47	Chr01 (54,709,107–54,718,953)	12,801
	**Heat shock protein 47**									
57	chHSP47	X57157.1	CAA40444	3,279	1,218	405	8.07	44.17	Chr01 (196,648,339–196,655,176)	8,890
	**Heat shock protein family B (small)**									
58	chHSP1	NM_205290	NP_990621	736	582	193	5.57	21.54	Chr19 (4,403,293–4,405,185)	1,893
59	chHSP2	XM_015297973	XP_015153459	1,501	543	180	5.57	20.31	Chr24 (6,238,971–6,244,063)	5,093
60	chHSP3	XM_001231557	XP_001231558	2,360	450	149	4.89	16.81	ChrZ (16,438,249–16,440,609)	2,361
61	chHSP7	XM_427836	XP_427836	1,000	453	150	5.93	16.34	Chr21 (4,315,538–4,317,144)	1,067
62	chHSP8	XM_004934409	XP_004934466	1,567	594	197	5.05	21.60	Chr15 (10,005,536–10,008,560)	3,933
63	chHSP9	NM_001010842	NP_001010842	993	582	193	5.35	20.62	Chr27(7,654,562–7,655,554)	993
64	chHSP11	NM_001277613	NP_001264542	907	426	141	4.58	15.61	Chr08 (25,462,690–25,466,126)	3,437
65	chHIKESHI	XM_015280837	XP_015136323	946	477	158	5.6	17.28	Chr01 (191,772,895–191,781,798)	11,574
66	chHSP25	AB154518	BAD34492	918	519	172	6.05	20.62	Chr09 (7,654,562–7,655,554)	1,291
67	chHSP10-1	AF031309	AAB86581	632	309	102	8.11	11.08	Chr07 (10,214,101–10,235,996)	28,464
68	chHSPE1	NM_205067	NP_990398	632	309	102	8.11	10.95	Chr07 (10,214,101–10,235,996)	28,464
69	chHSPB7L	XM_003641769	XP_003641817	727	492	163	4.82	17.33	Chr09 (16,075,864–16,077,249)	1,386
70	chLOC772158	XM_003642832	XP_003642880	885	603	200	5.16	21.81	Chr27 (7,653,016–7,653,900)	1,151
	**Heat shock protein family H (Hsp105/110)**									
71	chHSPH1	XM_015277924	XP_015133410	3,251	2,439	812	4.96	91.19	Chr01 (176,342,852–176,364,944)	28,721
72	chHSP108	AF387865.1	AAK69350.1	2,439	2,388	795	4.78	89.47	Chr01 (54,709,107–54,718,953)	12,801
73	chHSP105	XP_417113	NP_001153170	3,338	2,571	856	4.93	95.95	Chr01 (176,342,852–176,364,944)	28,721
	**Heat shock factor protein**									
74	chHSF3	NM_001305041	NP_001291970	3,637	1,656	551	4.6	60.62	Chr03 (282,366–297,374)	19,511
75	chHSF2	NM_001167764	NP_001161236	2,356	1,692	563	4.49	62.58	Chr03 (61,387,823–61,409,039)	21,217
76	chHSF1	NM_001305256	NP_001292185	1,774	1,476	491	4.92	53.47	Chr02 (130,889,950–131,021,873)	131,924

We identified 13 genes of the small HSP (sHSP) gene family, which contained at least one intact alpha-crystallin domain (HSP20) as confirmed by the Pfam and SMART tools. The 44 genes of the HSP40 gene family were subclassified based on domain conservation into types I, II, and III. In total, 11 HSP40 genes were classified as HSP40 type II (lack zinc-finger-like motifs); 29 as HSP40 type III due to the presence of a single J domain; and four (chDNAJA1, chDNAJA2, chDNAJA3, and chDNAJA4; 397–506 amino acids) as HSP40 type I, because they possessed the four characteristic canonical domains J, Glycine-phenylalanine(G/F)-rich region, two zinc-finger like motifs, and the carboxyl-terminal, first observed in *E. coli* (Table 1). Although 23 out of 36 chicken chromosomes contained chHSP40 genes, with the highest number of HSP40 genes found on chromosome 1, no HSP40 gene was found on chromosomes 16–18, 22, 24–26, 28–32, or W (Table 1).

**Fig. 1:**
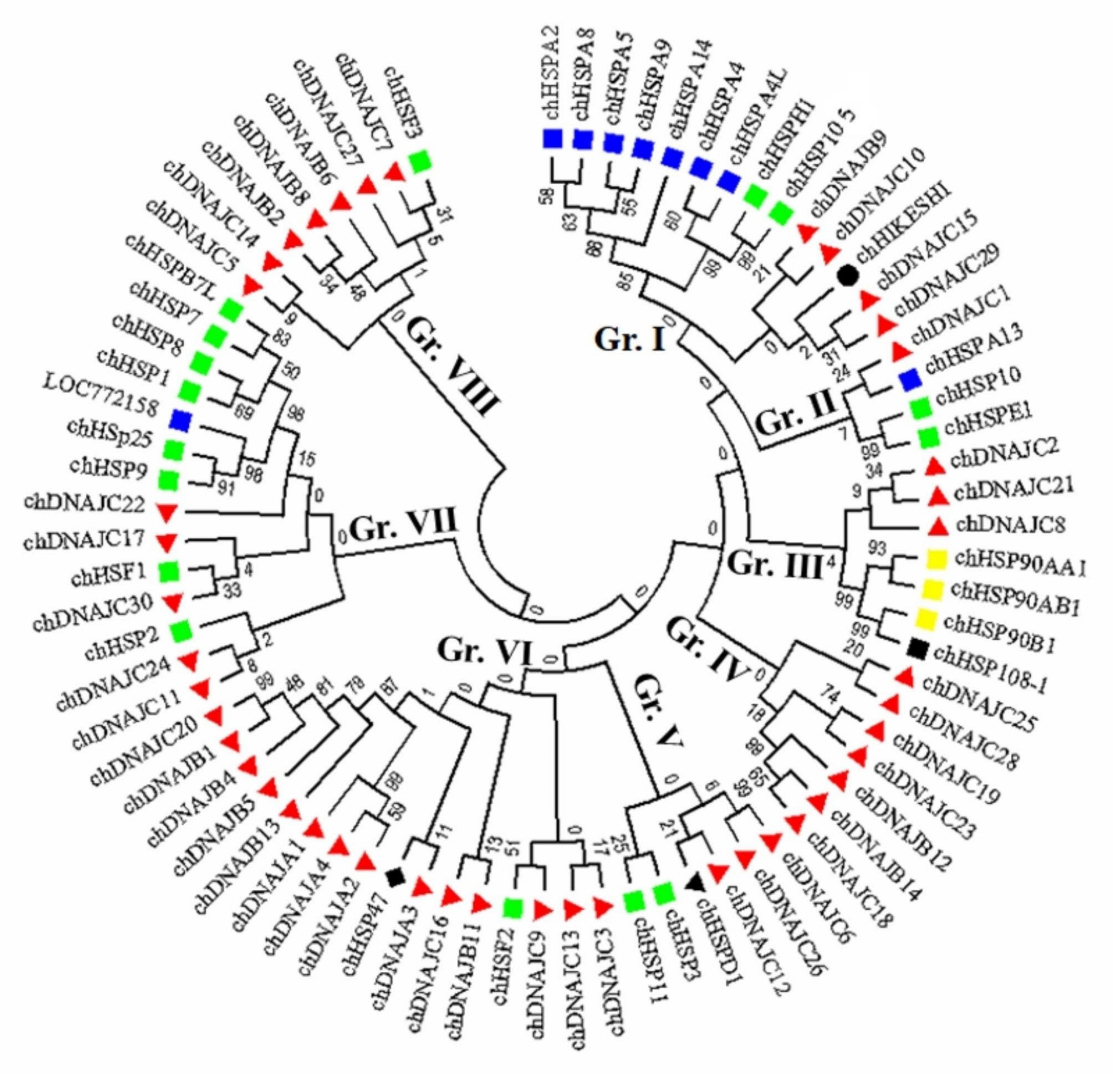
Phylogenetic analysis of chicken HSP genes. Full-length amino acid sequences of 76 chicken HSP genes were aligned using the BioEdit V.07 tool, and the phylogenetic tree was constructed in MEGA7 using the neighbor-joining method with 1000 bootstrap replicates

A total of 8 HSP70 genes along with their chromosomal positions are presented in Table 1. The genes chHSPA13 and chHSPA14 were mapped to chromosome 1; chHSPA4 and chHSPA9 to chromosome 13; and chHSPA4L, chHSPA2, chHSPA5, and chHSPA8 to chromosomes 4, 5, 17, and 24, respectively. Similarly, three genes were assigned to the HSP90 gene family; chHSP90AA1, chHSP90AB1, and chHSP90B1 were mapped to chromosomes 5, 3, and 1, respectively (Table 1). Three genes, chHSPH1, chHSP105, and chHSP108, were assigned to HSP105/108 gene family and mapped to chromosome 1. Three heat shock factor proteins of the HSF gene family, chHSF1, chHSF2, and chHSF3, were assigned to chromosomes 2 (chHSF1) and 3 (chHSF2–3; Table 1). Considering the chromosomal locations of all HSP genes analyzed in this study, chromosomes 16, 18, 22, 25–26, and 28–32 of the chicken genome completely lack HSP genes. To analyze the evolutionary relationships among HSPs in chicken, the protein sequences of the 76 HSPs were used to construct a phylogenetic tree, providing a new perspective for the classification of chicken HSPs. Based on the phylogenetic analysis, chicken HSPs were divided into eight clades (I–VIII; Fig. 1).

### Gene structure, domain organization, and physicochemical analysis of chicken HSPs

The physicochemical parameters indicated that the pI of small HSPs ranged from 4.49 to 11.01, with most members of this family being acidic except for chHSP10 and chHSPE1, which were found to be basic (Table 1). The pI levels of chicken HSP40 proteins ranged from 4.66 (chDNAJC24) to 11.01 (chDNAJC30; Table 1). Overall, 23 HSP40 proteins were acidic, while 21 were basic. In the HSP70 family, all members were acidic, with pI levels ranging from 5.03 (HSPA5) to 6.04 (HSPA13; Table 1). Similar results were obtained for the members of the HSP90 family, with chHSP90B1 having the lowest pI (4.75) and chHSP90AA1 the highest (4.92). HSP105/108 and HSF proteins were also acidic, with pI values ranging from 4.6 to 4.96 (Table 1). The HSP47 protein was basic (pI, 8.07), and the chicken HSP60 was acidic (pI, 5.6; Table 1).

**Fig. 2:**
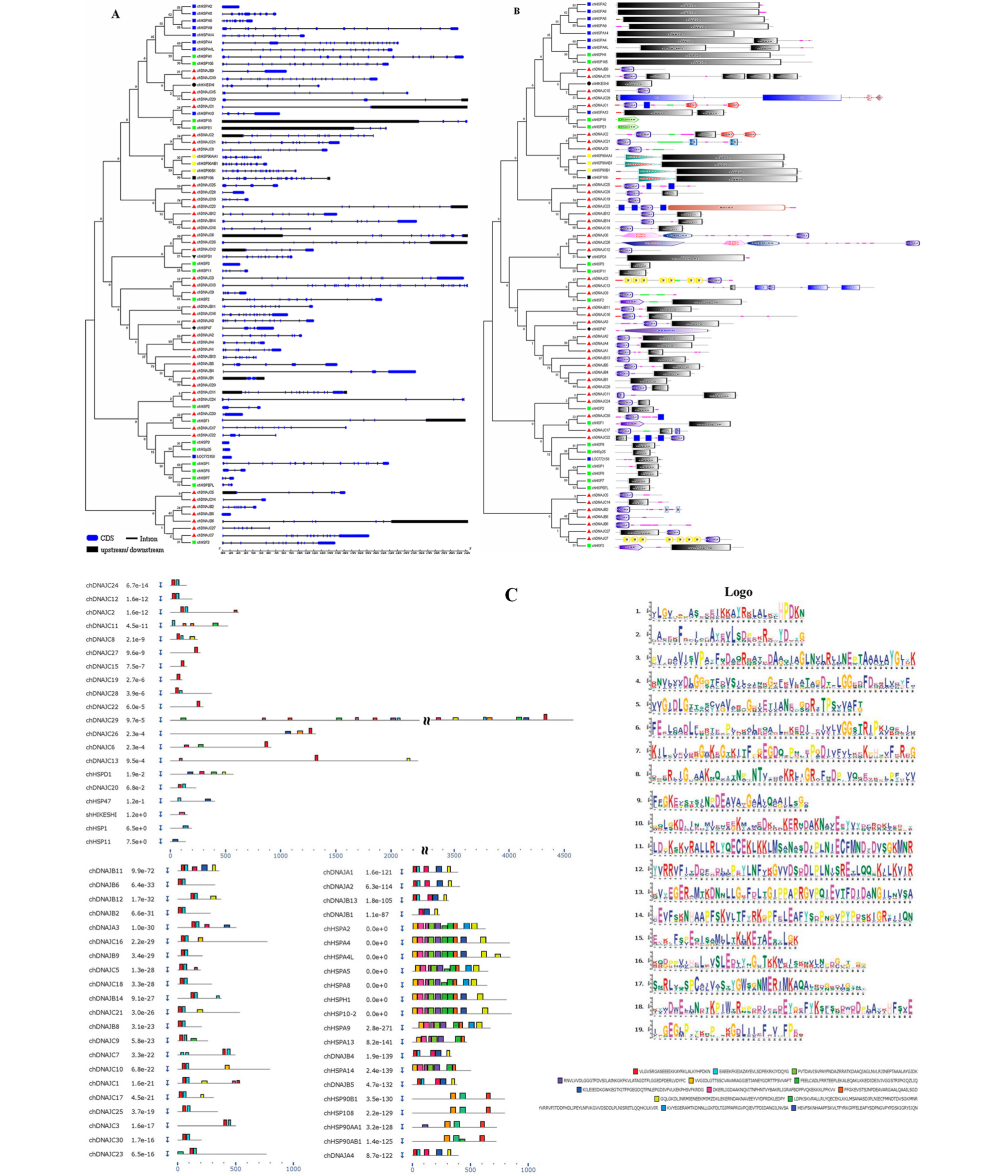
(**A**) Phylogenetic analysis and gene structures of chicken HSP genes. The unrooted neighbor-joining (NJ) tree was generated in MEGA7 with parameter settings indicated in Fig. 1 and based on full-length amino acid sequences of chicken HSPs. The solid green boxes represent exons, black boxes represent genes up/downstream, and black lines represent introns. (**B**) Phylogenetic analysis and domain architectures of chicken HSP genes. The unrooted neighbor-joining (NJ) tree was generated in MEGA7 with parameter settings indicated in Fig. 1 and based on full-length amino acid sequences of HSPs in chicken. (**C**) Conserved motifs of the chicken HSP genes were identified by MEME (http://meme-suite.org/). Grey lines represent non-conserved sequences, and each motif is indicated by a colored box numbered at the bottom. The lengths of the motifs in each protein are proportional

We used the chicken genome to analyze the gene structures of chicken HSP genes for a better understanding of the evolutionary conservation of this family. The exon-intron structures of the chicken HSP genes are shown in Fig. 2A. The numbers of introns/exons vary significantly among HSP genes, potentially indicative of functional diversity. However, the paralogous gene pairs derived from phylogenetic analysis shared a similar gene structure, and the number of introns in these genes ranged from none to 30 (Fig. 2A). Based on the number of introns, genes could be divided into two models: model 1 with no introns and model 2 containing more than one intron. Five of the 76 HSP genes, chHSP2, chHSP3, chHSP9, LOC772158, and chDNAJB8, were assigned to model 1. Model 2 was typical and contained the remaining 71 genes.

In order to be able to respond to various forms of stress in a timely manner, genes must be rapidly activated, which would be assisted by composed of functional units of protein. In this study, we used the SMART tool for the identification and annotation of protein domains and for the analysis of protein domain architectures (Fig. 2B). The proteins of HSP40 family usually had the DNAJ_CXXCXGXG domain, which contained four cysteine-rich repeats of the motif CXXCXGXG and was embedded in the N-terminus of DNAJ domain except in cases of chDNAJC29 and chDNAJC14. On the other hand, some proteins in the HSP40 family included multiple tandem tetratricopeptide repeats domain [24], which is a structural motif present in a wide range of proteins and mediates protein-protein interactions. It can couple with various domains to perform diverse functions as exemplified by chDNAJC3 and chDNAJC7 (Fig. 2B). The genes in the HSP70 family contained a single or double Pfam HSP70 domain, which mediates protein interaction with other signaling proteins in response to stress or disease. Finally, the other genes in the HSP family contained at least one Pfam HSP domain, which plays an important role in the activation of HSPs or induction of HSP gene expression (Fig. 2B).

To understand the functional diversification of the chicken HSP gene family, a conserved motif analysis was performed (Fig. 2C). We searched for 19 putative motifs in each gene. In general, HSP gene of the same group shared similar motifs. Motif 1 was present in most chicken HSP genes (70/76 HSPs), and motif 2 was present in 66 HSP genes. Seven HSPs, chDNAJC23, chDNAJC15, chDNAJC19, chDNAJC22, chHSP1, chHSP11, and chHIKESHI, had only one motif (Fig. 2C). Overall, the structure of the HSP proteins was conserved among the HSP family members.

### Potential regulatory relationship between chicken HSPs

**Fig. 3:**
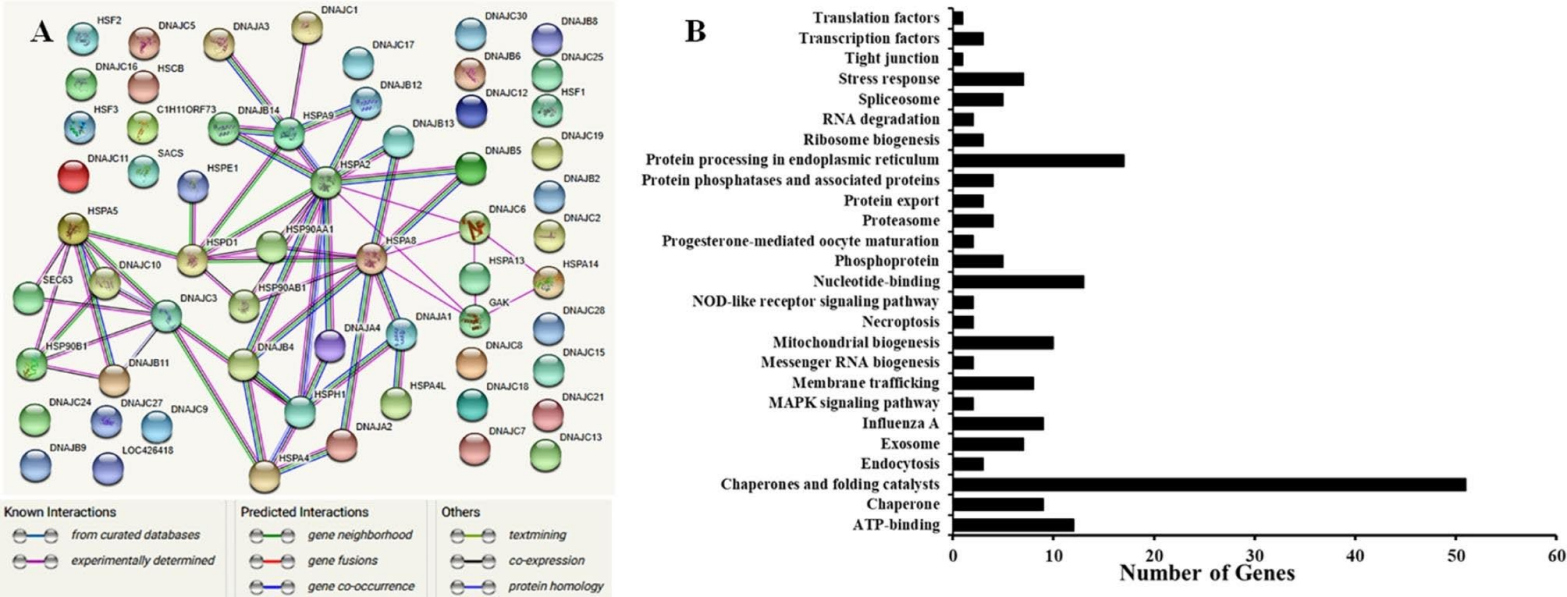
(**A**) Protein-protein interactions among chicken HSP proteins. Evidence-based medium-confidence interactions (score, 0.700) of HSPs with other HSPs were identified using the STRING program (https://string-db.org/). (**B**) Cellular pathways of proteins encoded by HSP genes. The cellular pathways of 76 proteins were determined using the KEGG pathway mapping database (https://www.genome.jp/kegg/) [45, 46]

Several studies have reported that HSPs in mammals are associated with or induce the expression of genes involved in various pathways including apoptosis, heat/cold stress, cytokine release, and immune response. Analysis of protein-protein interactions mainly helps in determining co-expressed and co-functioning proteins that cooperatively trigger or inhibit particular cellular functions [1, 2]. Interactions of HSPs with other HSP family members were examined using the STRING program (Fig. 3A). This analysis identified only 31 chicken HSP genes and could not identify the remaining 45 genes. Our results indicated strong interactions among chicken HSPs and identified more than 110 functional partners of chicken HSP genes. Specifically, the interactions between chHSP90B1 and chHSPA5 exhibited the highest scores (0.999 and 0.998, respectively) and those between chDNAJC3 and chDNAJB11 showed a high score (0.987 and 0.974, respectively). However, the major predicted interactions among HSPs were merely derived from text-mining data and experimental evidence, and lacked gene fusions. Therefore, co-expression and co-functional studies among HSPs should be conducted to provide experimental evidence.

For the cellular pathway analysis, we entered the 76 HSPs genes into the KEGG pathway-mapping database. The obtained pathway results were then integrated (Fig. 3B). Most HSP genes were associated with 26 pathways, and the major pathways were Chaperones and folding catalysts, Protein processing in the endoplasmic reticulum, Nucleotide-binding, ATP-binding, Stress response, Influenza A, and Mitochondrial biogenesis (Fig. 3B). Our results indicated that chicken HSP family genes mostly functioned in the regulation of gene expression and immune responses to pathogens.

### Expression profiles of HSP genes at different growth stages of chicken

**Fig. 4:**
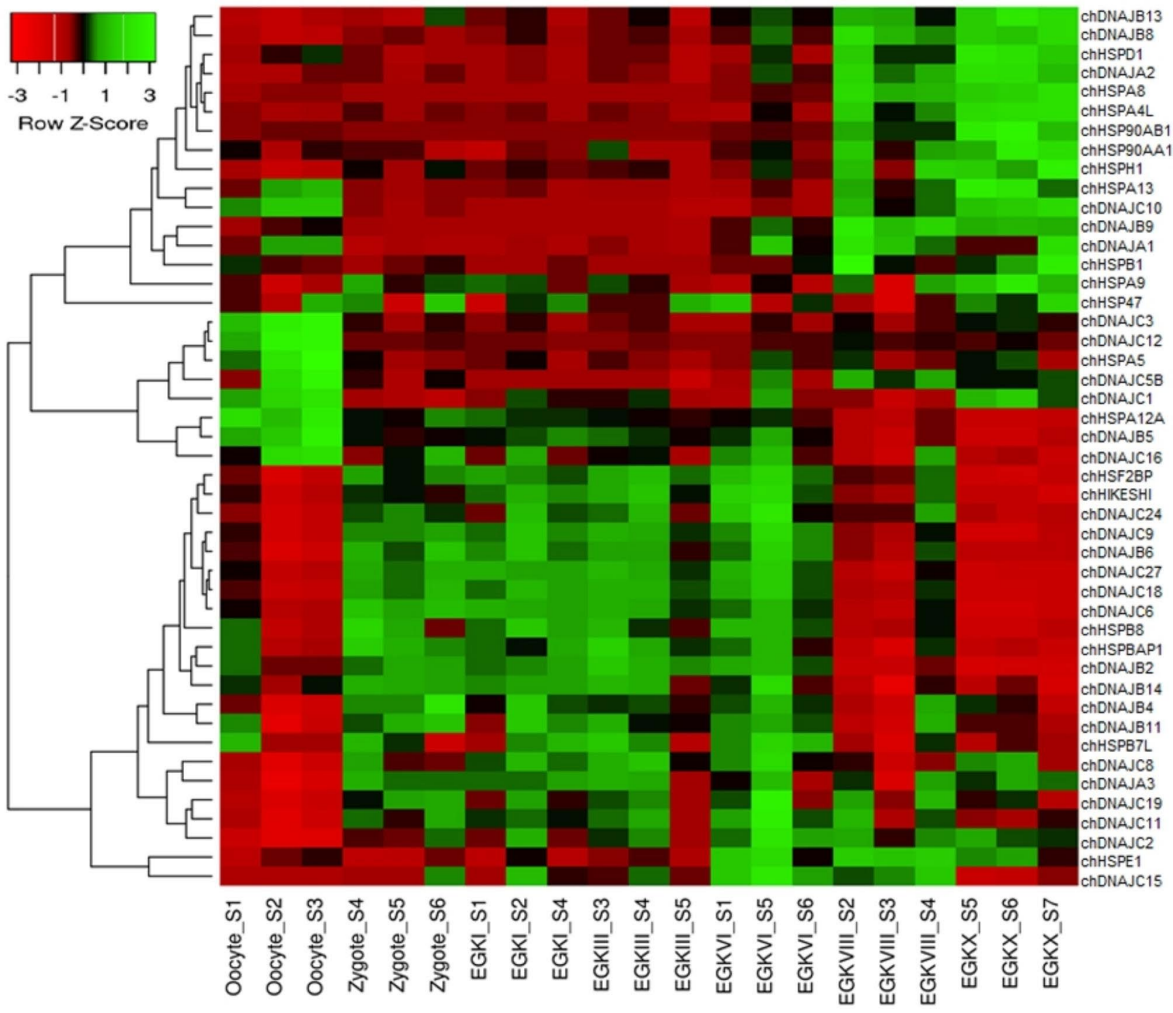
Expression profiles of HSP genes at 21 different growth stages of early chicken embryo, including the oocyte, zygote, and intrauterine embryos from Eyal-giladi and Kochav stage I (EGK.I) to EGK.X. Different colors correspond to log_2_-transformed values. Red and blue indicate higher and lower relative abundance, respectively

To gain insights into the temporal and spatial expression patterns of chicken HSP genes during embryonic development, the chicken RNA-seq data GSE86592 [25] were used to analyze the expression profiles of HSP genes at 21 different growth stages of chicken early embryo (Fig. 4). A total of 30 HSP genes were not expressed at any of the 21 growth stages. The results showed that 46 out of 76 HSP genes were expressed at all 21 growth stages of early embryonic growth at different levels. For example, chDNAJC3, chDNAJC12, and chHSP5 were upregulated in oocyte S1–3 stages but downregulated at all other growth stages (Fig. 4). Moreover, some groups of HSP genes were upregulated at some stages but downregulated at others. For example, chDNAJB8, chDNAJB9, chDNAJB13, chDNAJA2, chHSPD1, chHSPA4L, chHSPA8, chHSPH1, chHSPAB1, and chHSPAA1 genes were upregulated at EGKVIII_S2–4 and EGKX_S5–7 but downregulated at other stages of early embryonic growth (Fig. 4). In addition, chHSF2BP, chDNAJC9, chDNAJC18, chDNAJC27, chDNAJC6, chDNAJB2, and chDNAJB4 genes were upregulated at zygote S4-6, EGKI_S1–S4, EGKIII_S4, EGKIII_S5, EGKVI_S1, EGKVI_S5, and EGKVI_S6 and downregulated at other stages (Fig. 4). Our results indicated that the HSP genes were significantly expressed at early embryonic growth stages in chickens and also suggested that HSP genes were responsive to environmental conditions (Fig. 4).

### Expression profiles of HSP genes in indigenous Vietnamese ri chickens infected with HPAIV

To investigate the potential functions of HSP genes in response to HPAI infection, we analyzed HSP gene expression in the lung tissues of Vietnamese indigenous Ri chickens (both resistant and susceptible lines) infected with highly pathogenic H5N1 virus on post-infection days 1 and 3 by RNA-Seq analysis. We obtained 19.7–22.3 and 21.3–22.1 million sequence reads for the Ri resistant and susceptible chicken lines, respectively. Of the reads, 88.5–91.4% and 92.5–92.6% were successfully mapped to the chicken genome in cases of the Ri resistant and susceptible lines, respectively [26]. We obtained a total of 16,555 transcripts in HPAIV-infected resistant lines and 16,554 transcripts in susceptible ones. Similarly, we found a total of 16,555 and 16,554 transcripts in uninfected control resistant and susceptible lines, respectively, on day 1 and day 3 (data not shown). Then, differentially expressed transcripts (*P* ≤ 0.05; fold-change ≥ 2) were identified in HPAIV-infected resistant and susceptible lines compared with respective controls.

**Fig. 5:**
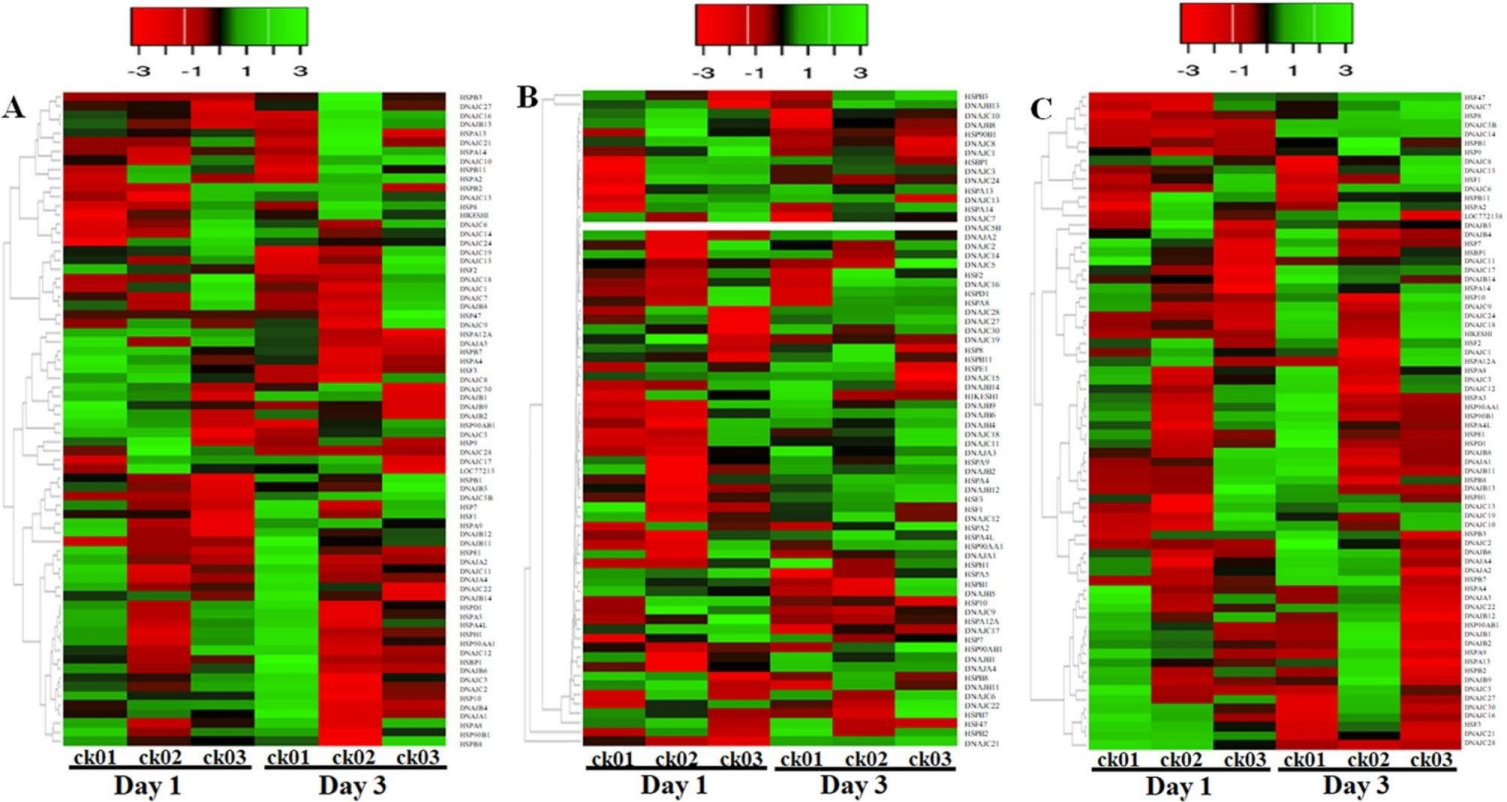
Expression profiles of HSP genes in two indigenous Ri chicken lines infected with HPAIV. RNA-seq data were used to produce heat maps. Red and green indicate high and low expression levels, respectively. (**A**) Expression profiles of chicken HSP genes in indigenous Ri resistant chickens infected with HPAIV compared to the controls. (**B**) Expression profiles of chicken HSP genes in indigenous Ri susceptible chickens infected with HPAIV compared to the controls. (**C**) Expression profiles of chicken HSP family genes in indigenous Ri resistant chickens infected with HPAIV compared to indigenous Ri susceptible chickens infected with HPAIV

The results of the transcriptome analysis of lung tissues of HPAIV-infected and control chicken lines are shown in Fig. 5A-C. Transcripts per million (TPM) values were used to express the transcript levels of chicken HSP genes. Our results showed that 72 of 76 HSP genes were expressed in lung tissues of the two chicken lines infected with HPAIV (Fig. 5A-C). Most chicken HSP genes exhibited broad expression patterns (Fig. 5A-C) with the exception of four genes (chDNAJC20, chDNAJC23, chDNAJC25, and chDNAJC26). In the resistant chicken line, four genes, DNAJC13, DNAJB13, DNAJB11, and DNAJC21, were significantly upregulated on day 3 of HPAIV infection (log_2_[fold change], 1.89–3.14) (Fig. 5A). In the susceptible line, four genes, DNAJB13, HSPB2, HSPB3, and DNAJB11, were dramatically upregulated on day 3 of HPAIV injection (Fig. 5B). The comparison of the resistant and susceptible chicken lines infected with HPAIV revealed nine genes that were significantly upregulated (DNAJB11, DNAJC13, DNAJC21, DNAJC5B, HSP47, HSP7, HSP9, and HSPB3; log_2_[fold change], 1.29–9.49), while four genes were significantly downregulated (DNAJB13, DNAJC14, HSP10, and HSPA2; log_2_[fold change], 1.075–7.67) on day 3 of HPAIV infection (Fig. 5C). Other HSP genes showed little change in expression levels after HPAIV infection in the two indigenous Ri chicken lines (Fig. 5A-C).

### Validation of RNA-Seq results by RT-qPCR

**Fig. 6:**
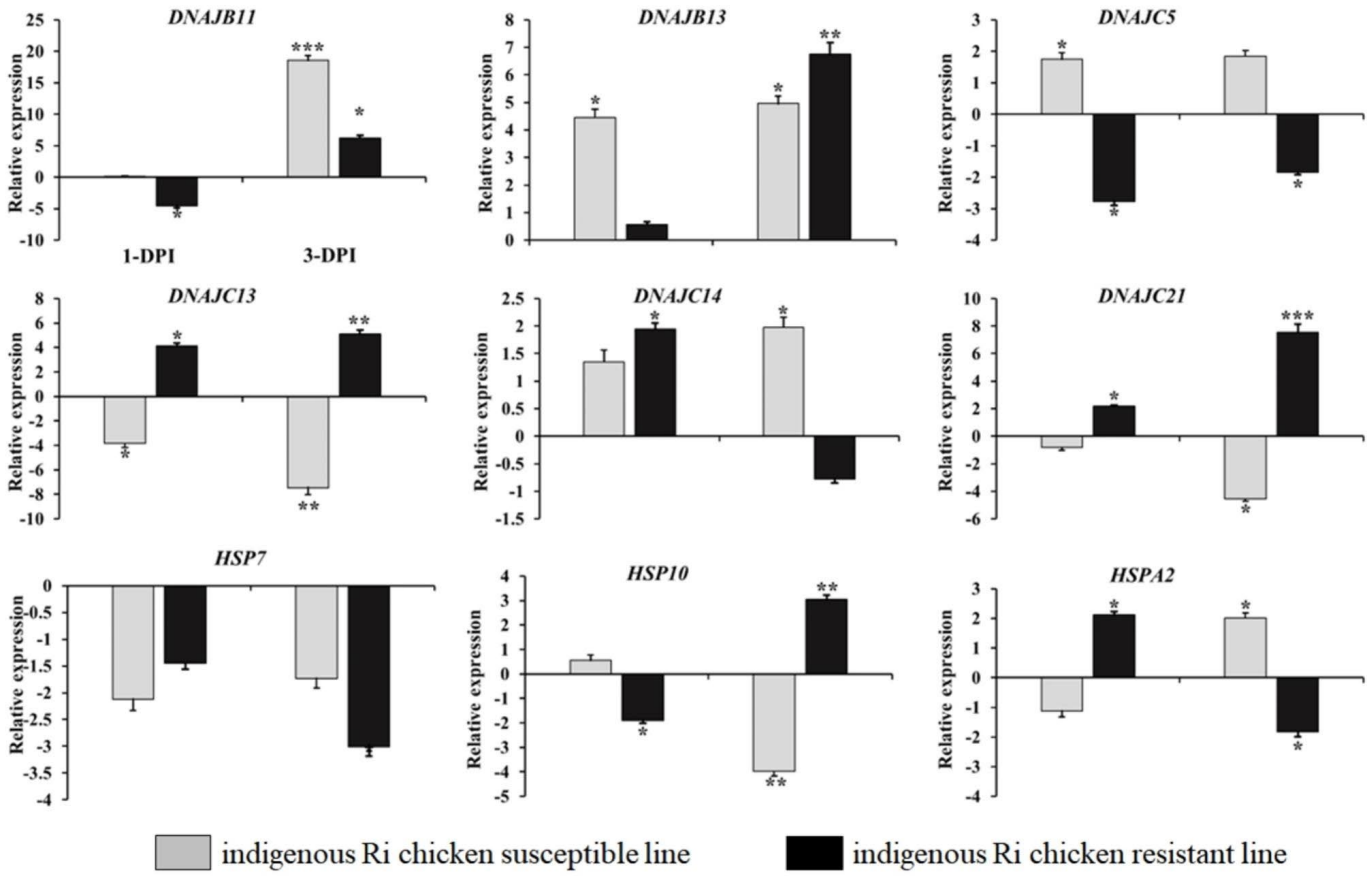
Validation of nine differentially expressed chicken HSP genes. The two indigenous Ri chicken lines were infected with 10^4^ EID50 of HPAIV. Lung tissues were isolated on post-infection days 1 and 3, and the transcript levels of indicated genes were determined by reverse transcription followed by quantitative PCR (RT-qPCR). Gene expression levels were normalized to those of *GAPDH*. Experiments were performed in triplicates on samples from five chickens. Significant differences in mRNA expression levels between two chicken lines are indicated as follows: *, *p* < 0.05; **, *p* < 0.01; and ***, *p* < 0.001. Error bars indicate SE of technical replicates (triplicates)

**Fig. 7:**
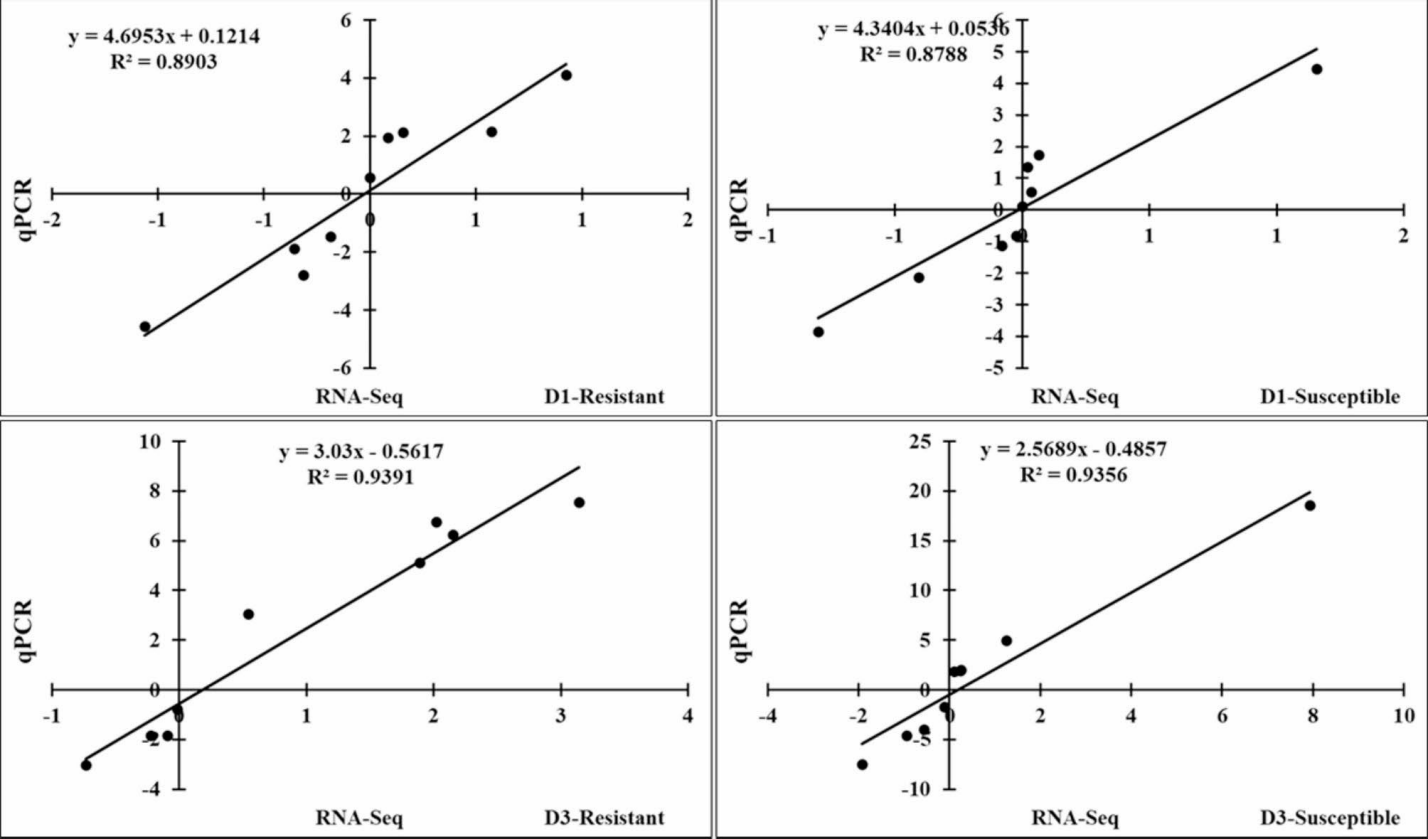
Significant correlations between the results of RT-qPCR and RNA-Seq analyses of gene expression in the lung of two indigenous Ri chicken lines infected with 10^4^ EID50 of HPAIV

To validate the RNA-Seq results, RT-qPCR was performed to analyze the expression levels of nine HSP genes in the lung tissues of the two indigenous Ri chicken lines infected with HPAIV compared with the respective uninfected controls (Fig. 6). The expression levels measured by RT-qPCR were consistent with the RNA-Seq results (correlation R^2^ = 0.8903 and 0.9391 for indigenous Ri resistant chickens infected with HPAIV at day 1 and day 3, respectively; and correlation R^2^ = 0.8788 and 0.9356 for susceptible chicken line infected with HPAIV at day 1 and day 3, respectively) as shown in Fig. 7. Generally, the accuracy of the RNA-Seq analysis was confirmed by the RT-qPCR.

## Discussion

Unlike previous studies focusing on one or several HSP families, we performed a genome-wide comprehensive survey of chicken HSPs, characterized all potential HSPs across the chicken genome, estimated potential regulatory relationships among them, analyzed transcriptional expression of HSPs at different stages of embryonic growth and in response to highly pathogenic avian influenza virus infection. Our analysis revealed a total of 76 genes (13 sHSP, 44 HSP40, 1 HSP47, 1 HSP70, 8 HSP70, 3 HSP90, 3 HSP105/110, and 3 HSF genes) in chicken. Chicken HSPs confer protection against a variety of cellular stressors [13] and are notably involved in cytoskeletal rearrangements [2] and apoptosis [9]. In this study, chicken HSP genes were observed to be dispersed over 23/36 chromosomes (Table 1), and similar results have been reported in humans [27], mice [19], fish [28], and plants [17, 29], suggesting the chicken HSP genes were conserved among species. Computational analysis of the physicochemical properties of a family of proteins helps in understanding their functions in vitro. In this study, 52 out of 76 chicken HSP proteins were observed to be acidic and 24 were basic, as indicated by their pI levels. These observations might be indicative of functional differences among chicken HSP proteins, as similar findings might suggest possibly different roles [2]. In any case, an in vivo assessment of chicken HSPs is necessary to understand their functional roles before making valid conclusions.

The phylogenetic, gene structure, and motif analysis of homologous sequences offer a wealth of information by identifying conserved residues crucial to the functions or structures of related proteins [30, 31]. Based on the phylogenetic, gene structure, and motif analysis of chicken HSPs, we found that the most closely related members of a family or subfamily exhibit similar exon/intron structures and intron numbers, and these results were also consistent with the characteristics defined in the above phylogenetic analysis. In the terminal branch of the phylogenetic tree, the numbers of exons/introns were very similar in some of the sister pairs. These findings indicated that some intron loss, along with intron gain events, might have occurred during the structural evolution of the chicken HSP genes. This pattern was also revealed by motif analysis; the type, order, and number of motifs in proteins of the same family/sub-family were similar but differed from those in proteins of other families and subfamilies.

Moreover, conservation of the structural architecture of HSP genes in humans, mice, plants, and fish has demonstrated that the N and C termini, though variable in sequence and length, are essential in preventing the misfolding of proteins; these observations were neatly validated by our findings [19, 28, 32]. HSP40 proteins are classified into three groups based on the presence of specific conserved regions. Type I DNAJ (DNAJA) proteins are characterized by four repeats of the CxxCxGxG-type zinc-finger region, a C-terminal region, and an N-terminal J-domain followed by a glycine/phenylalanine (G/F)-rich region. Type II DNAJ (DNAJB) proteins are very similar to DNAJA proteins, except that they lack the CxxCxGxG-type zinc-finger region. Type III DNAJ (DNAJC) proteins are the most diverse group, as they only carry the J-domain. The proteins that contain a J-like domain but lack the critical HPD tripeptide are classified as type IV J-proteins (DNAJD) [5, 16, 32] and functionally characterized based on their role as co-chaperones in binding and regulating the activities of HSP70s [19]. A total of 44 candidate chicken HSP40 genes were identified and found to be scattered across the chicken genome, and this number was higher than that in human genome with 41 HSP genes reported [28]. A large number of genes were identified in the HSP40 family that should be added to its functional mediatory role in stabilizing the interaction between HSP70 and many substrates in different cellular components to meet the functional of cellular [19, 28]. In addition, 23 (out of 44) chicken HSP40 genes appeared to be acidic based on their pI levels, and 21 exhibited basic properties, suggesting functional differences that may be useful in wet-lab experiments. DNAJ/HSP40 family members contain the J domain, facilitating their binding to HSP70s, although other domains that critical to their functioning have been identified [27]. A total of 8 members of HSP70 family were also identified in chicken with the invariant residues mostly found in the nucleotide-binding domain, where HSP70 proteins interact with the J domains of HSP40 proteins. Interestingly, all HSP70 proteins were predicted to be acidic with a minimal variation among their isoelectric points. This could suggest a functional similarity among the chicken HSP70s, further confirming the reports of conserved functional properties of HSP70 proteins across species [8, 21, 27].

In chicken, the HSP proteins play an important roles in apoptosis, heat stress response, environmental adaptation, immune response, and cell death, especially HSP70 proteins, though by associated or activated the signaling pathway or interact between HSPs family genes with or without signaling pathways [9, 13, 18, 33]. Our analysis demonstrated strong interactions among chicken HSPs, especially HSP70, HSP40, and HSP90 family genes/proteins. Recent research show that the HSP90 family genes play important roles in the etiology of several autoimmune diseases and various infections [7, 8]. The results also showed that the function of HSPs and their roles in molecular signaling and regulation of the immune system in chicken should be evaluated both in vitro and in vivo. Moreover, the remaining HSP proteins that have not been identified and characterized functionally in chicken should be investigated in future studies. Furthermore, the KEGG pathway analysis showed that the chicken HSP gene family is involved in apoptotic pathways, immune response to the pathogen, and signaling pathways [8]. The results indicated that the main functions of chicken HSPs were associated with the regulation of apoptosis, heat stress response, adaptation to environmental conditions, and immune response to infection.

Chicken RNA-Seq data from the public databases were further explored to dissect the expression profiles of the HSP genes analyzed in this study. Our analysis indicated that 30 HSP genes were not expressed in chicken early embryos at any of the 21 growth stages, while the remaining 46 genes were expressed at all 21 growth stages of chicken early embryos, with individual HSP genes showing different expression patterns during embryonic growth. Recent reports have indicated that HSP90, HSP70, and small HSP25 proteins play important roles in the development of embryonic chicken lens [16, 34]. Our results suggested that HSP genes play an important role at different growth stages of chicken early embryo. Moreover, 72/76 HSP genes were expressed in the lung tissues of the two indigenous Ri chicken lines (resistant and susceptible line) infected with HPAIV. The mRNA expression levels of DNAJB11, DNAJC13, DNAJC21, DNAJC5B, HSP47, HSP7, HSP9, and HSPB3 were significantly upregulated in lung tissues of HPAIV-infected chickens on day 3 of HPAIV infection, while those of DNAJB13, DNAJC14, HSP10, and HSPA2 mRNA were significantly downregulated. The expression levels of other HPS genes showed little change after the HPAIV infection. Recent reports indicate that HSP70, small HSP, HSF, HSP90, and HSP60 family proteins play important roles in response to heat stress and environmental conditions [9, 13, 14, 23]. Our study is the first to analyze the expression of HSP family genes in response to HPAIV infection in chickens, and the results suggest that HSP genes have essential functions in the development of the chicken embryo. The functions of the HSP genes in adult chickens, however, has been not investigated and should be examined in the near future. Furthermore, the remaining HSP proteins not characterized here should be investigated in future studies, especially to delineate the functions of HSP family genes in protection against infectious diseases in chicken.

## Conclusion

In this study, a total of 76 HSP genes in chicken were identified in a genome-wide survey and classified into 8 groups based on the phylogenetic tree analysis. Protein-protein interactions between HSP proteins were low, and their functional roles in chicken have not been characterized and should be investigated in a future study. On the other hand, KEGG analysis indicated that the chicken HSP gene family is involved in the regulation of apoptotic, heat stress, and immune response pathways. Finally, HSP genes were found to be differentially expressed in early chicken embryos and two HPAIV-infected indigenous Ri chicken lines. Our results will contribute to our understanding on the biological evolution of HSPs in chicken, with possible implications in animal breeding, and further sheds light on intercontinental chicken adaptation mechanisms.

## Materials and methods

### Identification of the HSP family members in chicken

In this study, we used the National Center for Biotechnology Information (NCBI) eukaryotic genome annotation resource database to search for the genome-annotated chicken *HSP* family genes. The NCBI chicken genome annotation release 103 (*Gallus gallus*, NCBI Annotation Release 103) contained several gene/protein isoforms. As multiple isoforms represent one common gene/protein, we selected only the first isoform and corresponding protein for further analysis. On the other hand, DNAJ protein was used as a keyword to search for J-proteins, and all candidate proteins were identified using the SMART database (http://smart.embl-heidelberg.de/). The retrieved sequences were translated using the online ORF finder tool (http://www.ncbi.nlm.nih.gov/gorf/gorf.html). Further, the predicted ORFs were verified using the BLASTP tool against the NCBI nonredundant protein sequence database. A total of 76 putative HSPs were identified in the chicken genome.

### Gene structures, domains, and phylogenetic analysis of chicken HSP genes

The gene structures of chicken HSP genes were analyzed using the Gene Structure Display Server (http://gsds.cbi.pku.edu.cn/). Protein-conserved motifs were identified using the MEME software as previously described [31] with the following parameters: optimum width, 10–60; the number of repetitions, any; maximum number of motifs, 15. The isoelectric point (pI) was calculated using the Compute pI/Mw tool (https://web.expasy.org/compute_pi/). The domain organizations of the HSP family proteins were analyzed using the SMART (http://smart.embl-heidelberg.de/), protein family (Pfam) (http://pfam.xfam.org/), and NCBI Bath Web CD-Search (https://www.ncbi.nlm.nih.gov/cdd) databases. The phylogenetic tree of HSP genes was constructed based on protein sequence alignment of the 76 HSP genes in chicken using the MEGA7 software with 1000 bootstrap resampling. The chicken HSP genes were classified into different groups based on the topology of the phylogenetic tree.

### Highly pathogenic avian influenza (HPAI) virus

Highly pathogenic avian influenza virus (HPAIV), A/duck/Vietnam/QB1207/2012 (H5N1), was used in this study. The viral isolate was propagated in 10-day-old embryonated chicken eggs at 37 ℃ for 48 h. The allantoic fluid (AF) of the eggs was then harvested, and aliquots of the AF were stored at − 80 ℃ until use, according to the OIE guideline (Chap. 3.3.4) [35]. The 50% egg infectious dose (EID50) of the influenza virus was determined as previously described [36]. Briefly, 10-fold serial dilutions of the virus were prepared in PBS, and 100 μl of each dilution was inoculated into the chorioallantoic cavities of 10-day-old embryonated chicken eggs. The eggs were incubated at 37 ℃ for 96 h. Five eggs were infected with each virus dilution. Harvested AF was tested for HA activity using 0.5% RBC according to the OIE guideline (Chap. 3.3.4) [35]. Calculation of the EID50 of virus suspension was performed using Reed and Muench mathematical technique [37].

### Indigenous Ri chicken infection with HPAIV

The specific-pathogen-free HPAIV resistant and susceptible indigenous Ri chicken lines (4 weeks of age) were purchased from the Poultry Research Centre of the National Institute of Animal Science, Vietnam. We conducted genotype analyses on Mx and BF2 to identify traits related to resistance and susceptibility as previously described [26, 38]. For the Mx protein, chickens carrying the 631-allele polymorphism A were selected as resistant, while those with the 631-allele polymorphism G were designated as susceptible. Similarly, among the BF2 haplotypes, B21 was associated with resistance, and B13 indicated susceptibility [26]. Consequently, chickens possessing the Mx(A)/BF2(B21) genotype were designated as HPAIV-resistant Ri chickens, whereas chickens with the Mx(G)/BF2(B13) genotype were categorized as HPAIV-susceptible Ri chickens. Infection of the two Vietnamese indigenous Ri chicken lines (resistant and susceptible) was conducted and performed according to the Animal Diseases-Diagnosis procedure (Ministry of Agriculture and Rural Development Vietnam, TCVN8400-26:2014). The protocols and guidelines were approved by the the Institutional Animal Care and Use Committee of the National Institute of Veterinary Research, Vietnam. The study was carried out in compliance with the ARRIVE guidelines. A total of 15 Ri resistant or susceptible chicken per group received intranasal inoculation of AF containing 10^4^ EID50 of A/chicken/Vietnam/NA-01/2019 (H5N1) in 200 μL solution. Uninfected resistant or susceptible Ri chickens served as controls. All Ri chickens were housed in single-layer cages, with each cage containing five Ri chickens. These cages were fitted with two food dispensers and a water trough. To prevent any potential spore interference, the control and treated groups were kept in separate poultry houses. All Ri chickens had free access to their respective diets and water. The chicken coops were cleaned twice daily, ensuring good air circulation. An insulation lamp maintained the indoor temperature around 25˚C. Following the viral infection, the chickens were monitored for clinical signs of disease, and the samples were collected on days one and three after infection, following the WHO Manual on Animal Influenza Diagnosis and Surveillance [39].

### Expression profiles of HSP genes in chicken at different growth stages using publicly available RNA-Seq data

To explore the expression profiles of *HSP* genes in chicken at different growth stages, including the oocyte, zygote, and intrauterine embryos from Eyal-giladi and Kochav stage I (EGK.I) to EGK.X, the public high-throughput RNA-seq data of chicken available in the Gene Expression Omnibus (accession number: GSE86592) submitted by Hwang et al. [25] were analyzed. A decision tree-based classification analysis was performed based on the class labels. Log2 trimmed mean of M value normalized values were used to calculate a gene expression matrix and consider library size in each sample by edgeR [25]. Spearman’s correlation coefficients were used to generate a distance matrix to characterize the linear relationship between class labels and gene expression. Hierarchical cluster analysis of the genes was performed using Cluster version 4.49 (http://www.bram.org/serf/Clusters.php) and the Java TreeView tool (http://jtreeview.sourceforge.net/). Cluster map analysis of the HSP genes detected at different growth stages was carried out using Euclidean distance. The *p* values were calculated using the right-tailed Fisher’s exact test, and those lower than 0.01 were considered to indicate significant differences.

### RNA extraction and quality analysis

Total RNA was extracted from lung tissues using the TRIzol RNA extraction kit (Invitrogen, Carlsbad, CA, USA), purified using the RNeasy Mini Kit (Qiagen, Germantown, MD, USA), and treated with DNase I (Promega, Madison, WI, USA) according to the manufacturer’s instructions. The RNA concentration and quality were further determined using an Agilent 2100 bioanalyzer (Agilent Technologies, San Diego CA, USA) and a Tecan F2000 microplate reader (Tecan Group Ltd., Männedorf, Switzerland). Samples with RNA integrity values greater than 7 and high-quality RNA (28 S/18S > 1) were used in the experiments [26].

### RNA‑sequencing (RNA‑seq) analysis

Potentially existing sequencing adapters and raw quality bases in the raw reads were trimmed using Skewer ver 0.2.2 [40]. The cleaned high-quality reads were mapped to the reference genome using the software STAR ver 2.5 [41]. Since the sequencing libraries were prepared using Illumina’s strand-specific library preparation kit, the strand-specific library option, --library-type = fr-first strand, was applied in the mapping process.

To quantify the mapped reads, Cufflinks ver 2.2.1 [42, 43] with the strand-specific library option, --library-type = fr-first strand, and other default options were used. The gene annotation of the reference genome gg6 from the UCSC genome (https://genome.ucsc.edu) in GTF format was used to obtain gene models, and the expression values were calculated in Fragments Per Kilobase of transcript per Million fragments mapped (FPKM). The genes differentially expressed between the two selected biological conditions were analyzed by Cuffdiff software in the Cufflinks package [42, 44] with the strand-specific library option, --library-type = fr-first strand, and other default options. To compare the expression profiles among the samples, the normalized expression values of the selected few hundred differentially expressed genes were clustered by in-house R scripts.

### Pathways and interaction analysis

The cellular pathways associated with the chicken HSP genes were analyzed using the Kyoto Encyclopedia of Genes and Genomes (KEGG; https://www.kegg.jp/kegg/) pathway-mapping database against organism-specific parameters (*Gallus gallus; gga*) [45, 46]. The interactions of chicken HSP proteins were analyzed by the Search Tool for the Retrieval of Interacting Genes/ Proteins (STRING, Version 10), as previously described [47]. The STRING database facilitates the analysis of gene/protein interactions in an organism-specific manner using commonly available sources, including the NCBI PubMed literature database. Moreover, functional annotation in the form of GO was subsequently extracted from the database using Blast2GO v2.7.1 (http://www.geneontology.org/).

### Quantitative real-time PCR (RT-qPCR) analysis of HSP transcripts

For cDNA synthesis, up to 3 μg of RNA was treated with 1.0 unit of DNase I and 1.0 μL of 10x reaction buffer (Thermo Fisher Scientific, Waltham, MA, USA) and incubated for 30 min at 37 ℃. Subsequently, 1.0 μL of 50 mM EDTA was added, and the mixture was incubated at 65 ℃ for 10 min to inactivate DNase I. Reverse transcription was performed then reverse transcribed using a Maxima First Strand cDNA Synthesis Kit (Thermo Fisher Scientific), according to the manufacturer’s recommendations. Primers were designed using the Lasergene software (DNASTAR Inc., Madison, WI, USA) and synthesized by PhuSa Genomic Co. Ltd. (CanTho, Vietnam; Table 2). Quantitative PCR was performed using a 2x Ampigen SYBR® Green Master Mix (Enzo Life Sciences, Farmingdale, NY, USA) according to the manufacturer’s instructions on a QuantStudio 5 Real-Time PCR System (Thermo Fisher Scientific). Gene expression levels were calculated using the 2^−ΔΔCt^ method and normalized to those of chicken *GAPDH* [48]. All RT-qPCR experiments were performed in triplicates.

**Table 2:** Primers used for quantitative PCR (qPCR) validations

Primer	F/R	The nucleotide sequence (5′-3′)	Accession No	Product Length (bp)
GAPDH	F	TGC TGC CCA GAA CAT CAT CC	NM_204305	
	R	ACG GCA GGT CAG GTC AAC AA		
DNAJB11	F	GGC CGC GTC TGT CTT CTC T	XM_422682	118
	R	GGT ACG CCT TCT TGA TGT CCT T		
DNAJB13	F	CCT CAC GCC CGA CAA GAA	XM_417251	117
	R	AAT ACC GGG GAA CGC TCA CT		
DNAJC5	F	ACG TGC TGG GAC TGG ACA AG	NM_001278008	114
	R	TTT CTG CCG CCT CTG GAT TA		
DNAJC13	F	TGC TGG GCT CAA GGT ATG G	XM_004939445	115
	R	TAA GCG TAG CAA GGT CAG TCT C		
DNAJC14	F	ACA GGC AGG CAG CAG AGG AGT	XM_001232018	127
	R	CAG GGG GAG GTG GGA AGC		
DNAJC21	F	TCT CTA CTG CCC TGC TTG TGA C	XM_004937181	83
	R	TCT CCC GAT GCT TCT TTG A		
HSP7	F	CTG CCC GAG GAT GTG GAC	XM_427836	105
	R	GAC GTG CTC GCT GTG CTT AG		
HSP10	F	GCG CAG CAG AGA CCG TAA C	AF031309	97
	R	GCT CCC GAT CCA ACT GCT A		
HSPA2	F	GGC CTT CAC CGA TAC AGA GC	NM_001006685	109
	R	ATA CTT GCG GCC GAT GAG AC		

### Statistical analyses

Statistical analysis was performed using the IBM SPSS software (SPSS 25.0 for Windows; SPSS, Chicago, IL, USA). The results are expressed as the mean ± standard error (SE) of three independent experiments for each group (n = 3) and were compared between groups using Duncan’s multi-comparison method.

## Data Availability

The datasets generated during the current study are available in the National Agricultural Biotechnology Information Centre (NABIC, http://nabic.rda.go.kr/) repository, under the IDs NN-5790-000001, NN-5793-000001, NN-5795-000001, NN-5794-000001, NN-5791-000001, NN-5792-000001, NN-5796-000001, and NN-5797-000001.

## List of abbreviations

Ch: Chicken
DNAJ: DnaJ Heat Shock Protein Family Member
HPAI: Highly pathogenic avian influenza
HS: Heat Shock
HSF: Heat shock factor
HSP: Heat shock protein
KEEG: Kyoto Encyclopedia of Genes and Genomes
qRT-PCR: Quantitative Real-Time PCR
RNA-seq: RNA-Sequencing
TPM: Transcripts per million
